# Disparate metabolic response to fructose feeding between different mouse strains

**DOI:** 10.1038/srep18474

**Published:** 2015-12-22

**Authors:** M. K. Montgomery, C. E. Fiveash, J. P. Braude, B. Osborne, S. H. J. Brown, T. W. Mitchell, N. Turner

**Affiliations:** 1 Department of Pharmacology, School of Medical Sciences, UNSW Australia, Sydney, NSW, Australia; 2 School of Health Sciences, University of Wollongong, Wollongong, NSW, Australia; 3 Illawarra Health and Medical Research Institute, University of Wollongong, Wollongong, NSW, Australia

## Abstract

Diets enriched in fructose (FR) increase lipogenesis in the liver, leading to hepatic lipid accumulation and the development of insulin resistance. Previously, we have shown that in contrast to other mouse strains, BALB/c mice are resistant to high fat diet-induced metabolic deterioration, potentially due to a lack of ectopic lipid accumulation in the liver. In this study we have compared the metabolic response of BALB/c and C57BL/6 (BL6) mice to a fructose-enriched diet. Both strains of mice increased adiposity in response to FR-feeding, while only BL6 mice displayed elevated hepatic triglyceride (TAG) accumulation and glucose intolerance. The lack of hepatic TAG accumulation in BALB/c mice appeared to be linked to an altered balance between lipogenic and lipolytic pathways, while the protection from fructose-induced glucose intolerance in this strain was likely related to low levels of ER stress, a slight elevation in insulin levels and an altered profile of diacylglycerol species in the liver. Collectively these findings highlight the multifactorial nature of metabolic defects that develop in response to changes in the intake of specific nutrients and the divergent response of different mouse strains to dietary challenges.

Obesity, insulin resistance and related metabolic health issues have escalated globally to epidemic levels. One of the major factors proposed to underpin this dramatic increase in the incidence of metabolic disease is excess caloric intake. While much of the focus in this area has been on increases in lipid intake, there is accumulating evidence that diets enriched in fructose (FR) also contribute to the risks of the metabolic syndrome^1^. Upon dietary intake, fructose is directly transported to the liver via the portal vein, where it is able to bypass several of the control steps of glucose metabolism (including phosphofructokinase), and therefore serves as an unregulated source of both glycerol 3-phosphate and acetyl-CoA^2^. The liver responds to flooding with acetyl-CoA by increasing lipogenic pathways and storing excess energy in form of triglycerides^3^. Ectopic lipid accumulation is further accelerated by elevated malonyl-CoA levels (the product of acetyl-CoA carboxylase), which inhibits CPT1, leading to reduced entry of fatty acids into mitochondria for oxidation^4^. Accordingly, excess hepatic lipid storage is a key characteristic observed in both rodents and humans after acute and long-term fructose intake^2^,^5^,^6^,^7^.

Elevated fructose consumption is also known to adversely impact whole body glucose tolerance and insulin sensitivity (summarised in^8^), however the mechanisms responsible for these effects are still not completely understood. While there are some discrepancies between different studies, livers from FR–fed rodents frequently exhibit (1) diacylglycerol (DAG) accumulation^9^,^10^,^11^, (2) activation of inflammatory and stress signalling pathways, including activation of c-Jun N-terminal kinases (JNK) and endoplasmic reticulum stress markers^12^,^13^,^14^,^15^,^16^,^17^ and (3) inhibition of components of the insulin signalling cascade^10^,^15^,^17^,^18^,^19^.

Mouse strains differ in their metabolic response to high calorie diets^20^,^21^,^22^,^23^,^24^, and we have recently shown that BALB/c mice are protected against high-fat diet-induced glucose intolerance and insulin resistance^24^. This favourable metabolic phenotype of BALB/c mice was strongly related to a lack of hepatic lipid accumulation^24^. Given that excess lipid deposition in the liver is a major factor driving metabolic dysfunction in response to FR-enriched diets, our goals in this study were to examine if BALB/c mice were also refractory to the metabolic defects induced by FR feeding and to additionally investigate the potential mechanisms connecting hepatic lipid accumulation with impaired glucose metabolism in this model.

## Results

### Body weight, adiposity and food intake

**Figure 1:**
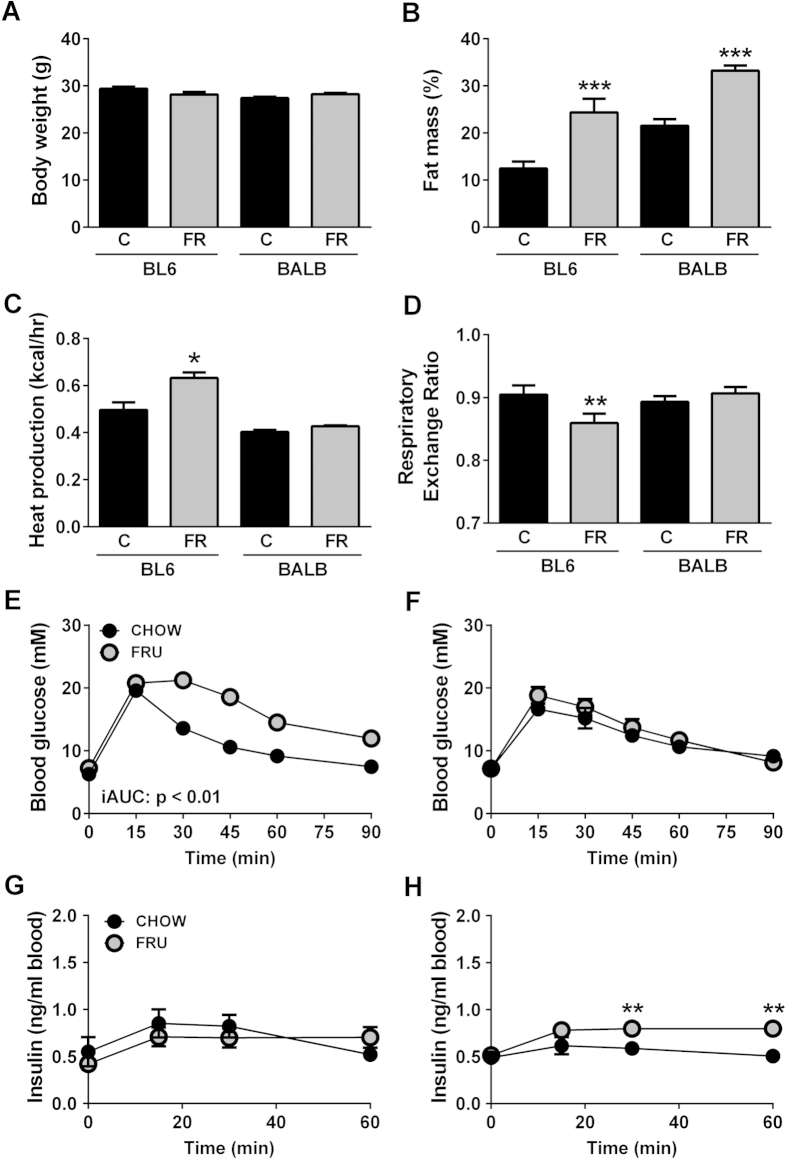
Metabolic characteristics in chow- and fructose-fed BL6 and BALB/c mice. Shown are (**A**) body weight, (**B**) fat mass, (**C**) energy expenditure (shown as heat production), (**D**) respiratory exchange ratio, as well as glucose tolerance curves in BL6 (**E**) and BALB/c (**F**) mice, and insulin levels during the GTT in BL6 (**G**) and BALB/c (**H**) mice. Black bars/dots = chow-fed mice, grey bars/dots = fructose-fed mice; shown are means ± SEM, with n = 26 (**A**), n = 8–12 (**B–H**); *p < 0.05, **p < 0.01, ***p < 0.001.

Body weight was unchanged in FR-fed BL6 and BALB/c mice when compared to chow diet (C)-fed control mice (Fig. 1A). Adiposity, indicated as percent fat mass (measured by EchoMRI) (Fig. 1B) and by the size of the epididymal, inguinal and retroperitoneal fat pads (Table 1), was increased in both mouse strains after FR-feeding. In contrast, liver and brown adipose tissue (BAT) weight was unchanged in FR-fed mice (Table 1). Food intake was measured weekly as an average of six cages with four mice per cage for each strain. Energy intake (in kcal/day/mouse) tended to be increased in both mouse strains on the FR-diet (+8.9% in BL6 mice and +8.6% in BALB/c mice), although this did not reach statistical significance.

### Circulating factors

In BL6 mice, plasma TAG were significantly decreased after FR-feeding and a similar trend was observed for non-esterified fatty acids (NEFA). In BALB/c mice, plasma TAG showed a similar reduction with FR-feeding, while plasma NEFA were significantly increased when compared to control BALB/c mice (Table 1). Fasting blood glucose and insulin levels remained unchanged with FR-feeding in both mouse strains (Table 1).

### Energy Expenditure and Fuel Selection

Energy expenditure was increased in FR-fed BL6 mice (+27%), but not in BALB/c mice, and ANCOVA analysis using (lean body mass +0.2x fat mass) as a covariate^25^ indicated this difference in BL6 mice was significant (Fig. 1C). The respiratory exchange ratio (RER) was decreased in BL6 mice but remained unchanged in FR-fed BALB/c mice (Fig. 1D).

### Glucose tolerance and Insulin levels

An intraperitoneal glucose tolerance test (GTT) was carried out as a measure of whole-body glucose clearance. FR-fed BL6 mice exhibited a substantial impairment in glucose clearance as evidenced by a significant increase in the incremental glucose AUC (+63%, Fig. 1E), while in contrast glucose tolerance was unaffected by FR-feeding in BALB/c mice (Fig. 1F). Blood insulin levels during the GTT were unchanged in FR-fed BL6 mice when compared to the control group (Fig. 1G), but were significantly increased at later time points in BALB/c mice (Fig. 1H).

### Ectopic lipid accumulation in the liver

**Figure 2:**
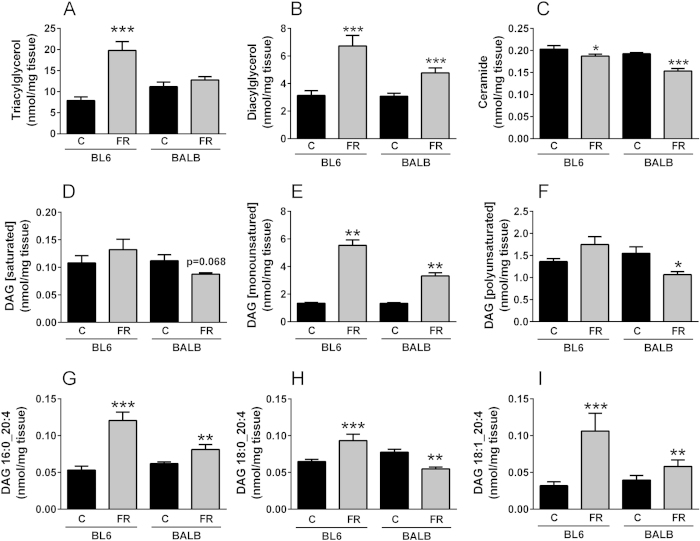
Hepatic lipid accumulation in chow- and fructose-fed BL6 and BALB/c mice. Shown are total triacylglycerol (**A**), diacylglycerols (DAG, **B**) and ceramide (**C**) levels, as well as hepatic content of saturated DAG (**D**), monounsaturated DAG (**E**) and polyunsaturated DAG (**F**), and accumulation of 16:0_20:4 (**G**), 18:0_20:4 (**H**) and 18:1_20:4 (**I**) DAG species. Black bars = chow-fed mice, grey bars = fructose-fed mice; shown are means ± SEM, with n = 4–5; *p < 0.05, **p < 0.01, ***p < 0.001.

Liver TAG were significantly increased in BL6 mice after FR-feeding (+225%), whereas excess TAG accumulation was absent in FR-fed BALB/c mice (Fig. 2A). These differences in hepatic lipid accumulation were unlikely due to differences in fructose utilization as fructokinase activity was increased to a similar extent in both strains after FR-feeding (BL6 C-diet 2.60 ± 0.34 FR-diet 5.07 ± 0.73*; BALB/c C-diet 2.16 ± 0.32 FR-diet 5.93 ± 0.64*** nmol/min/mg protein). Total diacylglycerol (DAG) content was increased in both FR-fed mouse strains when compared to controls (Fig. 2B), while in contrast there was a significant decrease in total ceramide (CER) content (Fig. 2C).

In addition, we observed striking differences in DAG species composition. Saturated (SFA) DAG species (saturated fatty acid at both the sn1 and sn2 position) remained largely unaffected by diet in both mouse strains (Fig. 2D), monounsaturated (MUFA) DAG species significantly increased in both strains (Fig. 2E), but it was the highly active polyunsaturated (PUFA) DAG species^26^ that exhibited strain-specific differences [DAG were classified as MUFA- or PUFA-DAG when at least one of two fatty acids was monounsaturated or polyunsaturated, respectively]. While total PUFA-DAG tended to increase in FR-fed BL6 mice (p = 0.062), they were significantly reduced in FR-fed BALB/c mice when compared to controls (Fig. 2F). Furthermore, 20:4-containing DAG species, that have previously been reported to potently activate protein kinase C (PKC)^27^, were either significantly decreased in FR-fed BALB/c mice but increased in BL6 mouse livers (18:0–20:4, Fig. 2H), or increased in both mouse strains, however to a much lesser extent in BALB/c mice (16:0–20:4, Fig. 2G, and 18:1–20:4, Fig. 2I).

### Lipid metabolism in the liver

**Figure 3:**
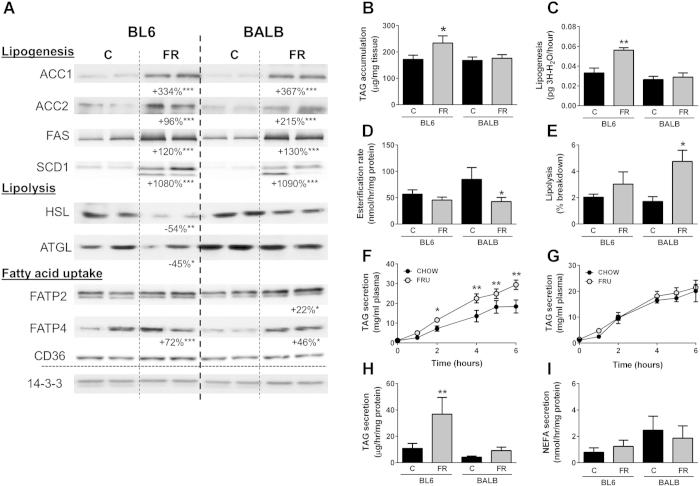
Lipid metabolism in livers and primary hepatocytes of chow- and fructose-fed BL6 and BALB/c mice. (**A**) Immunoblotting for lipogenic, lipolytic and fatty acid uptake proteins. Shown is representative immunoblotting with n = 2 per mouse strain and diet, however percentage changes and significance were calculated with n = 8 per group. ACC acetyl-CoA carboxylase, FAS fatty acid synthase, SCD1 stearoyl-CoA desaturase 1, HSL hormone-sensitive lipase, ATGL adipose triglyceride lipase, FATP2/4 fatty acid transport proteins 2/4, CD36 Cluster of Differentiation 36. 14-3-3 was used as a loading control. (**B**) Triglyceride accumulation in primary hepatocytes isolated from BL6 and BALB/c mice fed a chow- or fructose-diet for 2 weeks. (**C**) Lipogenic capacity of primary hepatocytes, measured as incorporation of [^3^H]H2O into the lipid pool, (**D**) Esterification rate of primary hepatocytes, measured as incorporation of 1-[^14^C]-palmitate into the lipid pool, and (**E**) lipolytic capacity, measured as the breakdown and oxidation of the [^14^C]-palmitate-labelled triglyceride pool. Furthermore, we measured hepatic triglyceride production and secretion rate using Poloxamer 407 over a period of 6 hours in BL6 (**F**) and BALB/c (**G**) mice, as well as triglyceride (**H**) and NEFA (**I**) secretion in primary hepatocytes. Shown are means ± SEM, with n = 4–5 mice (and 3 replicates per mouse) for the primary hepatocyte experiments, and n = 4 for the hepatic TAG secretion. Shown are means ± SEM, *p < 0.05, **p < 0.01, ***p < 0.001.

To investigate possible mechanisms for the observed differences in fructose-mediated ectopic lipid accumulation in the liver between BL6 and BALB/c mice, we took a comprehensive look at various hepatic lipid metabolism pathways, including lipogenesis, lipolysis, oxidation and lipid secretory pathways (Fig. 3). Consistent with the known effect of fructose to upregulate lipogenic pathways, protein contents of both acetyl-CoA carboxylase isoforms (ACC1 and ACC2), fatty acid synthase (FAS) and stearoyl-CoA desaturase (SCD1), were substantially elevated in FR-fed BL6 mice. Intriguingly, the protein levels of these lipogenic enzymes were also markedly upregulated in FR-fed BALB/c mice (Fig. 3A), despite the fact that TAG levels were unchanged. To determine if changes in protein content of lipogenic proteins correlated with changes in actual lipogenic capacity, we isolated primary hepatocytes from BL6 and BALB/c mice that were fed a chow- or FR-diet for 2 weeks, with subsequent overnight incubation of hepatocytes in FR-enriched media. This length of FR-feeding is sufficient to drive changes in lipogenesis in mice^13^,^15^. Similar to our observations in mouse liver, primary hepatocytes from FR-fed BL6 mice, but not BALB/c mice, showed increased TAG accumulation (Fig. 3B). Interestingly, lipogenesis, measured as the incorporation of [^3^H]H_2_O into the TAG pool, was significantly increased in FR-fed BL6 mice (in accordance with changes in ACC, FAS and SCD1), but remained unaffected by fructose exposure in BALB/c mice (Fig. 3C). Despite similar increases in the protein content of lipogenic markers in both mouse strains with FR-feeding, the tracer experiments suggested that BALB/c mice have a lower capacity to synthesise hepatic lipid from exogenous fructose. In addition to lipogenesis, a further mechanism known to affect hepatic lipid content is the breakdown of stored triglycerides, ie. lipolysis. The lipolytic enzymes adipose triglyceride lipase (ATGL) and hormone-sensitive lipase (HSL) were both significantly decreased in FR-fed BL6 mice when compared to control BL6 mice, but remained unchanged in BALB/c mice after FR-feeding (Fig. 3A), potentially pointing towards BALB/c mice being able to quickly degrade newly synthesised TAG. To verifiy the relevance of these immublotting results, we used [1-^14^C]palmitate to determine the esterification rate (= incorporation of exogenous FA into the lipid pool) and lipolytic capacity (=priming of the lipid pool with labelled palmitate, and subsequent determination of TAG breakdown) in primary hepatocytes. Whereas the esterification rate was reduced in FR-fed BALB/c mice (Fig. 3D), lipolytic capacity was significantly increased (Fig. 3E). In contrast, both measures were unaffected by FR-feeding in BL6 mice.

An additional process that can affect the total level of lipid storage in the liver is the extent of lipid export. To assess this we measured hepatic triglyceride secretion rate *in vivo* (using Poloxamer 407) in BL6 and BALB/c mice fed a FR-enriched diet for 8 weeks. Unexpectedly, FR-fed BL6 mice exhibited a significantly increased rate of TAG secretion (+67% at the 6-hour timepoint) when compared to control BL6 mice (Fig. 3F), whereas TAG secretion was unchanged in FR-fed BALB/c mice (Fig. 3G). These results are supported by a similar observation made in primary hepatocytes, with hepatocytes from FR-fed BL6 mice but not BALB/c mice showing a significantly increased rate of TAG secretion (Fig. 3H). In contrast NEFA secretion into the media from primary hepatocytes tended to be higher in BALB/c mice, but was unaffected by diet in both mouse strains (Fig. 3I).

Taken together, the results suggest that the lack of excess TAG storage in BALB/c mice in response to FR-feeding is partially explained by an altered balance between lipogenesis and lipolysis, as well as potentially reduced incorporation of exogenous FA into their hepatic lipid pool. This is interesting, as both mouse strains exhibit a moderate increase in the protein levels of some fatty acid uptake proteins, including fatty acid transport protein 2 (FATP2) and FATP4 (Fig. 3A).

### Oxidative metabolism in the liver

**Figure 4:**
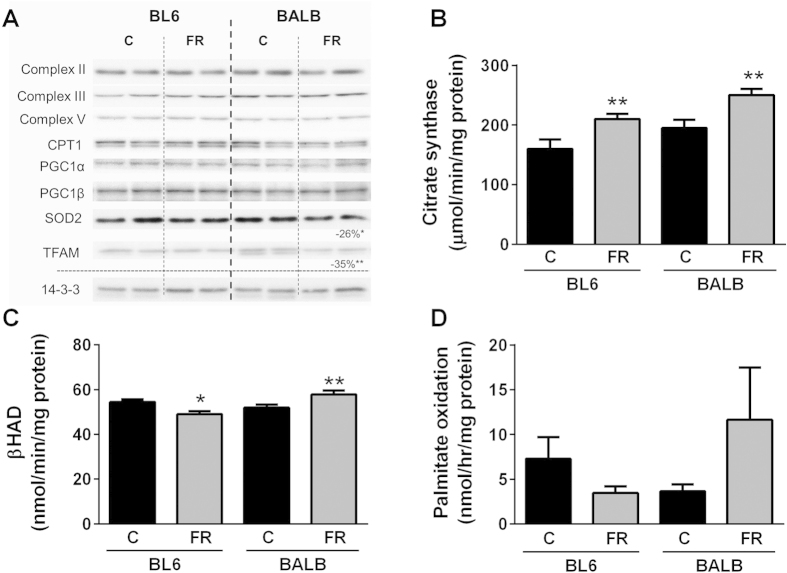
Oxidative metabolism in livers and primary hepatocytes of chow- and fructose-fed BL6 and BALB/c mice. (**A**) Immunoblotting for oxidative markers, including complexes II-V (subunits of complexes of the electron transport chain), CPT1 carnitine palmitoyl transferase 1, PGC1 peroxisome proliferator-activated receptor c coactivator 1, SOD2 superoxide dismutase 2, TFAM mitochondrial transcription factor A. 14-3-3 was used as a loading control. Shown is representative immunoblotting with n = 2 per mouse strain and diet, however percentage changes and significance were calculated with n = 8 per group. (**B**) Citrate synthase and (**C**) βHAD activity in liver homogenates, and (**D**) palmitate oxidation in primary hepatocytes. Shown are means ± SEM, n = 7–8 for liver homogenates and n = 4–5 mice (and 3 replicates per mouse) for the primary hepatocyte experiments, *p < 0.05, **p < 0.01.

In addition to the parameters measured above, changes in fatty acid oxidative metabolism may also contribute to the strain-specific differences in ectopic lipid accumulation in the liver. Whereas protein content of various oxidative markers, including subunits of the complexes of the electron transport chain (ETC), peroxisome proliferator-activated receptor gamma coactivator 1α (PGC1α), the mitochondrial transcriptional activator TFAM and carnitine palmitoyltransferase 1 (CPT1), remained largely unaffected by diet in both mouse strains (Fig. 4A), citrate synthase activity was significantly increased in both mouse strains after FR-feeding (Fig. 4B). Interestingly, βHAD (β-Hydroxy acyl-CoA dehydrogenase) activity (Fig. 4C) as well as palmitate oxidation (using [1-^14^C]palmitate in isolated hepatocytes) (Fig. 4D) were reduced in FR-fed BL6 mice, but in contrast were higher in FR-fed BALB/c mice when compared to controls, suggesting increased capacity to oxidize lipids in FR-fed BALB/c mice is another factor likely contributing to the absence of excess hepatic lipid accumulation in this mouse strain.

### PKC translocation in the liver

**Figure 5:**
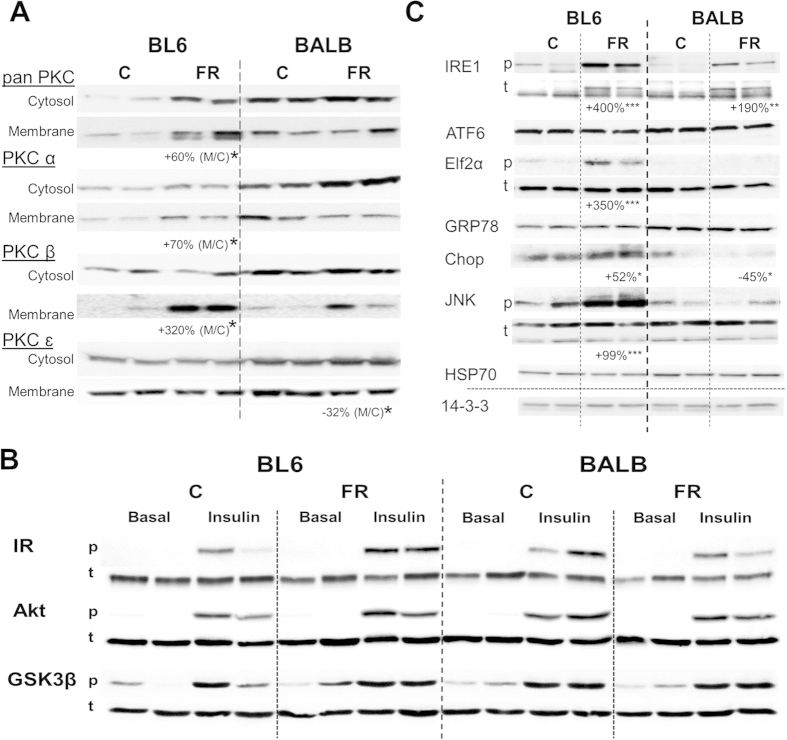
PKC activation, insulin signalling and markers of endoplasmic reticulum (ER) stress in livers of chow- and fructose-fed BL6 and BALB/c mice. (**A**) Cytosolic and membrane-associated content of protein kinase C isoforms. (**B**) Phosphorylation status of the insulin receptor (IR), the protein kinase Akt and glycogen synthase kinase 3β (GSK3β) was determined in livers after 8 weeks dietary intervention and 15 min of insulin stimulation at 2 U/kg lean mass. (**C**) Activation of ER stress pathways was determined as phosphorylation status of Elf2a (E74-like factor 2a), IRE1 (Inositol-requiring enzyme-1) and JNK (c-Jun N-terminal kinase), and protein content of ATF6 (activating transcription factor 6), GRP78 (78 kDa glucose-regulated protein), Chop (C/EBP homologous protein) and HSP70 (heat shock protein 70). 14-3-3 was used as a loading control. Shown is representative immunoblotting with n = 2 per mouse strain and diet, however percentage changes and significance were calculated with n = 5–8 per group. *p < 0.05, **p < 0.01, ***p < 0.001.

Differences in lipogenesis, lipolysis and fatty acid oxidative metabolism are potential mechanisms explaining strain-dependent differences in FR-induced hepatic TAG accumulation, however these differences are unlikely able to explain the retained glucose tolerance of FR-fed BALB/c mice. TAG are relatively inert lipid species, while it is thought that the more metabolically active lipid intermediates DAG and CER primarily affect glucose tolerance and insulin sensitivity^28^,^29^. The strain-dependent differences in DAG accumulation, particularly PUFA-DAG (Fig. 2), potentially suggested that alterations in this lipid class may partially contribute to the disparate effects of fructose on glucose tolerance, as DAG are known to stimulate the translocation of PKC isoforms to the plasma membrane which impair insulin signalling at the level of the insulin receptor (IR) and insulin receptor substrates (IRS)^29^,^30^. To determine if PKC translocation was differentially affected by FR-feeding in the two mouse strains, we carried out a fractionation procedure to separate the cytosolic and membrane compartments, and examined distribution of various PKC isoforms by immunoblotting. Using a pan-PKC antibody, we observed that both cytosolic and membrane levels of PKC remained unaffected by diet in BALB/c mice, while total PKC content increased in BL6 mouse livers after FR-feeding in both compartments (to a greater extent in the membrane fraction: +60% [membrane/cytosol]) (Fig. 5A). Furthermore, FR-fed BL6 mice exhibited a significant increase in the conventional protein kinases PKCα and PKCβ1 in the membrane fraction (Fig. 5A), while no change in translocation of these isoforms was observed in FR-fed BALB/c mice. In addition, translocation of the novel protein kinase PKCɛ was unchanged by diet in BL6 mice, but was significantly decreased in FR-fed BALB/c mice (Fig. 5A).

### Effects of fructose-feeding on insulin signalling and ER stress in liver

Despite increased activation of several hepatic PKC isoforms in FR-fed BL6 mice, insulin stimulations *in vivo* did not reveal any impairment in components of the insulin signalling cascade in the liver, including unchanged phosphorylation of IR, the protein kinase Akt and glycogen synthase kinase β (GSK3β) (Fig. 5B). Similarly, insulin signalling was unaffected by FR-feeding in livers of BALB/c mice (Fig. 5B). Endoplasmic reticulum (ER) stress has been implicated as an important mediator of insulin resistance^31^,^32^,^33^,^34^, and was also shown to be increased under high-fructose conditions in rodents^10^,^13^,^15^. To examine changes in ER stress after FR-feeding, we examined several ER stress markers in the liver by immunoblotting (Fig. 5C). Phosphorylation (=activation) of eukaryotic initiation factor 2α (Elf2α), inositol-requiring enzyme 1 (IRE1) and c-Jun N-terminal kinase (JNK) were significantly increased in FR-fed BL6 mice, whereas Elf2α and JNK phosphorylation were completely absent or unchanged in FR-fed BALB/c mice and IRE1 phosphorylation was increased to a lesser extent (+190% in BALB/c mice vs. +350% in BL6 mice). The expression of C/EBP homologous protein (CHOP) was significantly increased in FR-fed BL6 mice, but reduced in FR-fed BALB/C mice. Activating transcription factor 6 (ATF6), 78 kDa glucose-regulated protein (GRP78) and heat shock protein 70 (HSP70) protein levels were unaffected by diet in both mouse strains (Fig. 5C).

### Lipid metabolism, insulin signalling and ER stress in muscle

**Figure 6:**
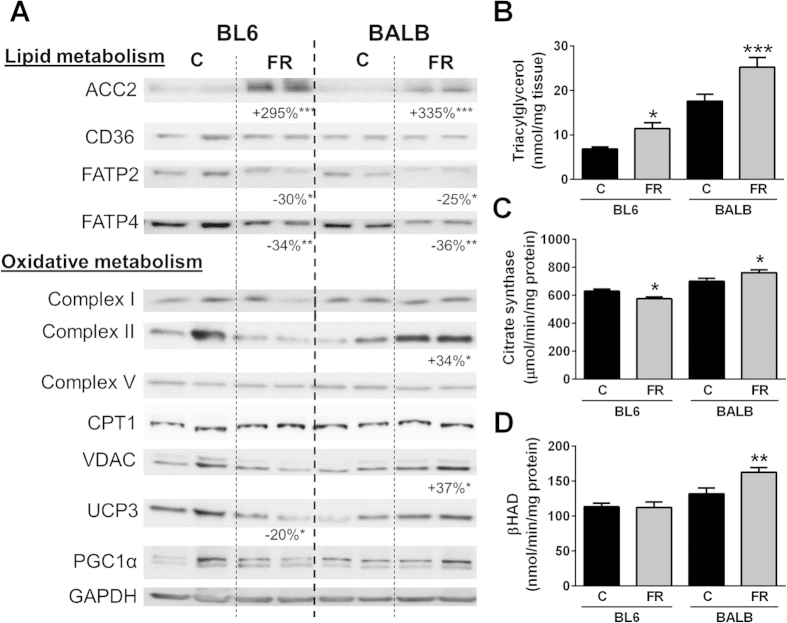
Lipid and oxidative metabolism in quadriceps muscle. (**A**) Immunoblotting results for markers of fatty acid uptake and oxidative metabolism. Shown is representative immunoblotting with n = 2 per mouse strain and diet, however percentage changes and significance were calculated with n = 8 per group. ACC acetyl-CoA carboxylase, CD36 cluster of differentiation 36, FATP2/4 fatty acid transport proteins 2/4, complexes II-V subunits of complexes of the electron transport chain, CPT1 carnitine palmitoyl transferase 1, VDAC mitochondrial outer membrane protein porin, UCP3 uncoupling protein 3, PGC1 peroxisome proliferator-activated receptor c coactivator 1. GAPDH was used as a loading control. (**B**) Muscle triglyceride content, (**C**) citrate synthase and (**D**) βHAD activity of muscle homogenates. Shown are means ± SEM, with n = 6–8 per group, *p < 0.05, **p < 0.01, ***p < 0.001.

**Figure 7:**
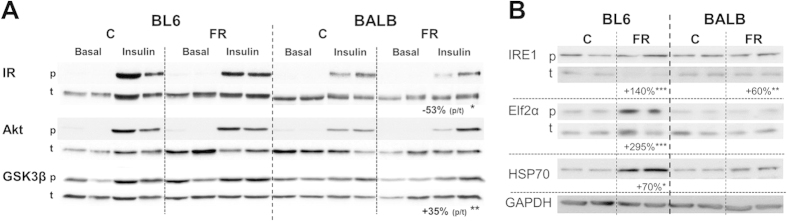
Insulin signalling and markers of endoplasmic reticulum (ER) stress in quadriceps muscle of chow- and fructose-fed BL6 and BALB/c mice. (**A**) Phosphorylation status of the insulin receptor (IR), the protein kinase Akt and glycogen synthase kinase 3β (GSK3β) was determined in livers after 8 weeks dietary intervention and 15 min of insulin stimulation at 2 U/kg lean mass. (**B**) Activtion of ER stress pathways was determined as phosphorylation status of Elf2a (E74-like factor 2a) and IRE1 (Inositol-requiring enzyme-1) and protein content of HSP70 (heat shock protein 70). GAPDH was used as a loading control. Shown is representative immunoblotting with n = 2 per mouse strain and diet, however percentage changes and significance were calculated with n = 5–8 per group. *p < 0.05, **p < 0.01, ***p < 0.001.

Whereas BALB/c mice exhibited profound protection against FR-induced ectopic TAG accumulation in the liver, this protection was not present in skeletal muscle, with substantial TAG accumulation in BALB/c mice, to an even greater extent than observed for FR-fed BL6 mice (Fig. 6B). Markers of lipid metabolism and oxidative capacity were assessed to determine the potential causes of TAG accumulation. In both strains there was a substantial increase in total ACC2 content after FR-feeding, while markers of fatty acid entry into muscle (measured as protein content of the fatty acid transport proteins CD36, FATP2 and FATP4) were reduced (Fig. 6A). The levels of various proteins involved in oxidative metabolism (subunits of ETC complexes, PGC1α, uncoupling protein 3 (UCP3) and voltage-dependent anion channel (VDAC, used as a marker of mitochondrial content)), exhibited only minimal differences between strains and diets (Fig. 6A). However, as also observed in the liver, we found discrepancy between the content of specific mitochondrial proteins and the activity of oxidative enzymes in skeletal muscle. In BL6 mice, FR-feeding led to a decrease in citrate synthase activity (Fig. 6C) with no change in βHAD activity (Fig. 6D), whereas in FR-fed BALB/c mice both oxidative proteins showed increased activity. In addition to hepatic insulin signalling, we also investigated changes in IR, Akt and GSK3β phosphorylation under insulin-stimulated conditions in muscle. While insulin signalling remained unaffected by FR-feeding in BL6 mice (as observed for liver), FR-fed BALB/c mice exhibited decreased phosphorylation of IR and GSK3β (Fig. 7A). Furthermore, in BL6 mice ER stress markers (phosphorylation of IRE1 and Elf2α, as well as HSP70) were significantly increased with FR-feeding, while in BALB/c mice only phosphorylation of IRE1 exhibited an increase when compared to controls (Fig. 7B).

## Discussion

This study highlights mouse strain-specific differences in the metabolic response to dietary intake of different nutrients and investigates possible mechanisms of fructose-induced metabolic deterioration. There is a large body of literature demonstrating that dietary intake of high amounts of fructose leads to fatty liver and the development of glucose intolerance and insulin resistance (reviewed in^8^). This metabolic phenotype was clearly present in BL6 mice, which exhibited increased adiposity, ectopic lipid accumulation in liver and muscle, and developed glucose intolerance following a FR-enriched diet. Interestingly, BALB/c mice did not exhibit any deterioration of glucose intolerance, despite increased adiposity and muscle lipid accumulation. This favourable metabolic phenotype was associated with a marked difference in hepatic lipid profile, including reduced TAG accumulation and a reduction in the levels of polyunsaturated DAG species (PUFA-DAG). The improved glucose clearance in FR-fed BALB/c mice was also associated with a reduction in hepatic ER stress markers and a mild elevation in circulating insulin levels during the GTT.

The DAG-PKC axis is frequently suggested as an important mechanism of lipid-induced insulin resistance in the liver, through antagonism of the insulin signalling cascade^29^,^30^. In BL6 mice the overall content of DAG was elevated and the content of PUFA-DAG tended to also increase with FR-feeding, with several 20:4-containing species being significantly elevated (16:0/20:4, 18:0/20:4 and 18:1/20:4). In BALB/c mice the increase in specific PUFA-DAG species was either blunted or even decreased after FR-feeding (especially DAG species 18:0/20:4 was reduced). *In vitro* comparisons of various DAG species and their ability to activate PKCs suggests that polyunsaturated DAG, and especially DAG containing arachidonic acid (20:4) are particularly effective at inducing PKC activation^26^,^27^,^35^. Indeed, the elevated 20:4-containing DAG species in FR-fed BL6 mice were associated with increased activation of PKCα and to an even greater extent of PKCβ1 after FR-feeding, whereas no activation of these PKC isoforms was observed in BALB/c mouse livers. The PKC isoform that has been most extensively linked to lipid-induced insulin resistance in liver is PKCɛ, with its activation frequently documented as a mechanism interconnecting hepatic DAG accumulation and insulin resistance (reviewed in^36^). We observed no increase in the activation of this PKC isoform in BL6 mice under our experimental conditions, and PKCɛ activation was mildly decreased in liver of FR-fed BALB/c mice. Despite the significant increase in the activation of the conventional PKC isoforms PKCα and PKCβ in FR-fed BL6 mice, hepatic insulin signalling was unaffected by FR-feeding in both strains in our study, potentially indicating that PKCɛ is indeed the critical hepatic PKC isoform that antagonises insulin signalling during the development of insulin resistance, as proposed previously^9^,^37^,^38^,^39^. It is also possible that in this experimental model, like others recently reported^40^,^41^,^42^,^43^, there is not a straight-forward relationship between insulin resistance and DAG accumulation/PKC activation. In muscle we also observed no impairment in insulin signalling that might explain the impaired glucose tolerance in FR-fed BL6 muscle, however DAG accumulation and PKC activation were not directly examined in this tissue. Collectively our findings suggest that in response to dietary fructose overload, factors beyond changes in insulin signal transduction might be more important regulators of glucose homeostasis.

Another factor linked to the development of insulin resistance is ER stress^34^. Upon increased ER stress, cells respond by activating three parallel but interconnected pathways of the unfolded protein response (UPR), the IRE1/XBP1 arm, the PERK/elf2α and the ATF6 arm, as an attempt to resolve the elevation in cellular stress^44^. In the present study, we have shown that FR-fed BL6 mice exhibit an induction of two arms of UPR, the IRE1/XBP1 arm as well as the PERK/elf2α arm. In contrast, in FR-fed BALB/c mice activation of the IRE1/XBP1 arm was blunted whereas signalling through the PERK/elf2α and the ATF6 arms was completely absent. ER stress is suggested to be a major contributor to the development of hepatic insulin resistance, by (1) increasing *de novo* lipogenesis and (2) activating the c-Jun N-terminal kinase (JNK) and IkB kinase (IKK) pathway and subsequently inhibiting hepatic insulin signalling^34^. Our results suggest that the variable response in ER stress signalling in response to FR-feeding in BL6 and BALB/c mice might be more closely related to changes in lipogenesis than insulin signalling, as neither strain showed a defect in hepatic insulin signalling, but we did observe increased lipid synthesis in hepatocytes from the BL6 strain, where ER stress markers were higher. A recent study in FR-fed mice treated with fenofibrate similarly showed that activation of ER stress markers correlates very closely with lipogenesis in the liver^10^. In addition to lipid synthesis, activation of the ER stress pathway has also been shown to affect glucose homeostasis by directly regulating gluconeogenic pathways and glucose tolerance^45^,^46^,^47^, and it is possible that the impairment in glucose tolerance in FR-fed BL6 mice in the current study might also be related to direct effects of cellular stress on gluconeogenesis and hepatic glucose output.

A further factor potentially contributing to some extent to the observed strain-specific differences in glucose tolerance was an alteration in circulating insulin during the GTT. FR-fed BALB/c mice exhibited a mild increase in insulin levels at some of the later points during the GTT, however this elevation was not seen in FR-fed BL6 mice. Fructose is not thought to have a major direct impact on insulin secretion^48^, although it has been reported that dietary fructose may change the insulin response to a subsequent glucose challenge in rodents^49^. It is currently unclear what underpins the strain-dependent difference in insulin response and whether the mild elevation in insulin levels in FR-fed BALB/c mice in the present study reflects altered insulin secretion or a change in insulin clearance in the liver.

The contrast in the accumulation of hepatic TAG between FR-fed BL6 and BALB/c mice was also intriguing, particularly since DAG levels were significantly elevated in both strains. The degree of lipid accumulation in the liver is the result of a complex interplay between fatty acid influx, *de novo* lipogenesis, fatty acid oxidation and lipid export pathways. In a recent report we showed that unlike other strains, hepatic TAG levels remained largely unchanged in BALB/c mice fed a high-fat diet compared to low-fat controls, with differences in fatty acid uptake proteins potentially explaining the disparate effects between strains^24^. An enhanced rate of lipogenesis is considered the primary mechanism underpinning hepatic lipid deposition with dietary fructose and, whereas both strains exhibited marked induction in the protein content of lipogenic enzymes such as ACC, FAS and SCD-1, lipogenic capacity in primary hepatocytes (determined as incorporation of [^3^H]-H_2_O into the lipid pool) was increased in FR-fed BL6, but not in BALB/c animals. In addition to lower lipogenic activity in FR-fed BALB/c mice, tracer work in primary hepatocytes also suggested increased lipolytic and oxidative capacity in this strain. In line with these findings, recent work has shown in skeletal muscle that direct upregulation of lipolysis by ATGL overexpression can lead to enhanced mitochondrial capacity and fatty acid oxidation^50^. Our findings indicate that the lack of hepatic lipid accumulation in BALB/c mice was likely due to adaptations in several metabolic pathways, and potentially suggest that newly synthesised fatty acids in FR-fed BL6 mice are channelled into TAG pools which are less readily broken down, while TAG in FR-fed BALB/c mice may be more readily degraded hence leading to minimal overall change in TAG content.

There is a substantial body of literature demonstrating that high fructose intake increases the risks of developing metabolic disease (reviewed in^1^). Comparing fructose-induced differences in metabolic phenotype in two commonly used mouse strains we have highlighted the multifactorial nature of metabolic defects that develop in response to altered intake of specific dietary nutrients. Additionally we have shown that while some metabolic adaptations to elevated fructose intake are common to C57BL/6 mice and BALB/c mice, there are also marked strain-specific differences, further emphasising the importance of considering the genetic background of mice when undertaking metabolic studies.

## Methods

Ten-week old C57BL/6J and BALB/c mice were purchased from the Australian Resource Centre (Perth, Australia). Mice were maintained in a temperature-controlled room (22 °C ± 1 °C) with a 12-hour light/dark cycle and *ad libitum* access to water and experimental diets^15^,^51^. After one week on a standard control “chow” diet (71% of calories from carbohydrate as wheat/starch, 8% calories from fat, 21% calories from protein, ~3kcal/g; Gordon’s Specialty Stock Feeds, Yanderra, NSW, Australia), mice were randomly allocated to remain on the chow diet (C) or to receive a home-made diet enriched in fructose (FR; 35% of calories from fructose, 35% calories from starch, 10% calories from fat, 20% calories from protein, ~3.1kcal/g) *ad libitum* for 8 weeks (or 2 weeks for primary hepatocyte experiments). All experiments were approved by the University of New South Wales Animal Care and Ethics Committee, and followed guidelines issued by the National Health and Medical Research Council of Australia.

### Body composition and energy expenditure

Fat mass was measured using the EchoMRI-900 Body Composition Analyser (EchoMRI Corporation Pte Ltd, Singapore) in accordance with the manufacturer’s instructions. Heat production and respiratory exchange ratio (RER) of individual mice were measured using an Oxymax indirect calorimeter (Columbus Instruments, Columbus, OH, USA) as previously described^52^.

### Glucose tolerance, insulin levels and *in vivo* insulin stimulations

Mice were fasted for 6 hours and then injected intraperitoneally with glucose (2 g/kg); blood glucose levels were monitored over time using an Accu-check II glucometer (Roche Diagnostics, Castle Hill, NSW, Australia). Fasting blood insulins as well as insulin levels during the GTT were measured using an Ultrasensitive Mouse Insulin Elisa Kit (Crystal Chem, Illinois, USA). For *in vivo* insulin stimulations, mice were fasted overnight and given an intraperitoneal injection of insulin at 2U/kg lean mass, with tissues rapidly excised and snap frozen 15 min after the injection.

### Tissue lipid analyses and lipid secretion

Tissue and media triglyceride contents were determined using a colorimetric assay kit (Triglycerides GPO-PAP; Roche Diagnostics, Indianapolis, IN, USA) as previously described^53^. Similarly, plasma NEFAs (and NEFAs in primary hepatocyte media) were measured using a colorimetric kit (WAKO diagnostics, Osaka, Japan). For diacyglycerol (DAG) and ceramide measurements, lipids were extracted in solvents^54^ containing 2 nmoles of ceramide (17:0) and 10 nmoles of DAG (17:0/17:0) as internal standards for quantification, and samples were analysed by mass spectrometry as described previously^24^. Hepatic triglyceride production rate was measured using Poloxamer 407 over a period of 6 hours, as described previously^55^.

### Primary hepatocyte isolation

Primary hepatocytes were isolated by collagenase perfusion. Briefly, liver was perfused through the inferior vena cava with EGTA buffer (HBSS buffer +0.5 mmol/l EGTA) for 15 min, followed by collagenase digestion (Collagenase H, Roche) in calcium buffer (HBSS buffer +2 mmol/l CaCl2) for 9 min. Hepatocytes were plated on collagenase-coated 6-well plates with 500,000 cells per well, firstly in adherence media (Gibco M199 media +100 U Penicillin/Streptomycin, 0.1% BSA, 2% FBS, 100 nmol/l Dexamethasone, 100 nmol/l Insulin) for 4 hours, then in basal media (Gibco M199 media +100 nmol/l Dexamethasone, 1 nmol/l Insulin) overnight, in the presence or absence of 2 mmol/l fructose in the media. For TAG and NEFA secretion into the media, basal media was collected after overnight incubation and TAG/NEFA levels are expressed per hour and mg protein.

### Analysis of lipogenic, lipolytic and enzyme activities

Lipogenic capacity was measured as the incorporation of [^3^H]H_2_O (0.2 mCi/ml) into the lipid pool over a 2-hour period. Lipids were extracted using the Folch procedure as described previously^56^ and incorporation of ^3^H into the lipid fraction was assessed. Measurement of the esterification rate and lipolytic capacity were set up in the form of a pulse-chase experiment. Hepatocytes were incubated in basal media (±fructose) in the presence of 200 μmol/l palmitate (conjugated to 1% BSA) and 1-[^14^C]-palmitate (2 μCi/ml) for a period of 2.5 hours. After the priming period, a subset of hepatocytes was analysed to determine the esterification rate (=amount of palmitate incorporated into the lipid pool). All other cells were washed, fresh media (±fructose) applied and the lipolytic capacity determined in a subsequent 1-hour incubation period. Lipolytic capacity was measured as the breakdown of labelled triglycerides and appearance of the [^14^C]-carbon in CO_2_ and the lipid and aqueous fractions in the media. In addition, we measured the ability of hepatocytes to oxidise exogenous 1-[^14^C]-palmitate. Cells were incubated in basal media (±fructose) in the presence of 200 μmol/l palmitate (conjugated to 1% BSA) and 1-[^14^C]-palmitate (0.5 μCi/ml) for one hour. At the conclusion of the assay the media was acidified with 1 mol/l perchloric acid and palmitate oxidation was determined as the accumulation of [^14^C]-carbon in the CO_2_ and the acid-soluble metabolite (ASM) fraction. Citrate synthase, βHAD and fructokinase activities were measured as described previously^52^,^57^.

### Liver fractionation

Liver fractionation was carried out as described previously^58^. Briefly, liver tissue was homogenised in 4 volumes of homogenisation buffer (20 mmol/1 MOPS, pH 7.5, 250 mmol/1 mannitol, 1.2 mmol/1 EGTA, 200 μg/ml leupeptin, 2 mmol/l benzamidine, and 2 mmol/1 phenylmethylsulfonyl fluoride (PMSF)) in a Precellys (Sapphire Bioscience, Waterloo, NSW, Australia) at 6 m/sec for 30 sec, followed by ultracentrifugation at 175.000 g and 4 °C for 15 min. The supernatant (=*cytosolic fraction*) was frozen for subsequent immunoblotting. The pellet was resuspended in homogenisation buffer, recentrifuged at the same speed, the supernatant discarded and the pellet resuspended in solubilisation buffer (20 mmol/1 MOPS, pH 7.5, 1% (vol/vol) Triton X-100,  mmol/1 EDTA, 2 mmol/1 EGTA, 200 μg/ml leupeptin, 2 mmol/1 benzamidine, and 2 mmol/1 PMSF). After nutation for 1 hour at 4 °C, the samples were centrifuged at 175,000 g and 4 °C for 15 min, and the supernatant (=*membrane fraction*) frozen for subsequent immunoblotting.

### Immunoblotting

Whole-tissue lysates were prepared from powdered quadriceps muscle and liver on a Precellys (Sapphire Bioscience Australia) at 6 m/sec for 30 sec, in RIPA buffer^59^. Proteins were resolved by SDS-PAGE electrophoresis and immunoblot analysis was conducted as described elsewhere^51^,^52^,^59^,^60^. Immunolabelled bands were quantitated using ImageJ 1.44p software.

### Statistical analysis

All results are presented as mean ± standard error. Data were analysed with an unpaired student’s *t*-test. Statistical significance was accepted at P < 0.05.

## Additional Information

**How to cite this article**: Montgomery, M. K. *et al.* Disparate metabolic response to fructose feeding between different mouse strains. *Sci. Rep.* **5**, 18474; doi: 10.1038/srep18474 (2015).

## Figures and Tables

**Table 1:** Food intake, tissue weights and circulating factors in chow- and fructose-fed C57BL/6 and BALB/c mice.

	C57BL/6		BALB/c	
	CHOW	FRU	CHOW	FRU
Food Intake (kcal/mouse/day)	10.60 ± 0.55	11.82 ± 0.41	8.83 ± 0.32	9.76 ± 0.35
Liver (%)	4.22 ± 0.20	4.18 ± 0.16	4.83 ± 0.16	4.54 ± 0.08
Epi.WAT (%)	1.18 ± 0.06	1.42 ± 0.11*	2.41 ± 0.13	3.04 ± 0.12**
Ing.WAT (%)	0.93 ± 0.04	1.14 ± 0.05**	1.87 ± 0.21	2.21 ± 0.08***
Retro.WAT (%)	0.26 ± 0.03	0.35 ± 0.03*	0.97 ± 0.06	1.27 ± 0.06**
BAT (%)	0.24 ± 0.01	0.26 ± 0.01	0.36 ± 0.03	0.37 ± 0.03
Plasma TAG (μmol/ml)	2.04 ± 0.23	1.56 ± 0.26*	1.86 ± 0.19	1.30 ± 0.09**
Plasma NEFA (μmol/ml)	0.62 ± 0.05	0.48 ± 0.05*	0.47 ± 0.04	0.60 ± 0.03*
Fasting Glucose (mM)	8.44 ± 0.49	7.55 ± 0.28	7.73 ± 0.36	7.01 ± 0.17
Fasting Insulin (ng/ml)	1.17 ± 0.30	0.92 ± 0.12	1.05 ± 0.07	1.10 ± 0.09

Tissue weights are expressed as ‘percent of body weight’. Epi.WAT epididymal white adipose tissue; Ing.WAT Inguinal white adipose tissue; Retro.WAT retroperitoneal white adipose tissue; BAT brown adipose tissue; TAG triacylglycerol; NEFA non-esterified fatty acids. Shown are means ± SEM, n = 7–8 for each measure, *p < 0.05, **p < 0.01, ***p < 0.001.
